# A Stillborn Multiple Organs’ Investigation from a Maternal DENV-4 Infection: Histopathological and Inflammatory Mediators Characterization

**DOI:** 10.3390/v11040319

**Published:** 2019-04-02

**Authors:** Priscila Nunes, Rita Nogueira, Janice Coelho, Francisco Rodrigues, Natália Salomão, Carollina José, Jorge de Carvalho, Kíssila Rabelo, Elzinandes de Azeredo, Rodrigo Basílio-de-Oliveira, Carlos Basílio-de-Oliveira, Flávia dos Santos, Marciano Paes

**Affiliations:** 1 Laboratório de Imunologia Viral, Instituto Oswaldo Cruz, Fundação Oswaldo Cruz, Rio de Janeiro 21040-900, Brazil; pricgn@ioc.fiocruz.br (P.N.); elzinandes@ioc.fiocruz.br (E.d.A.); flaviab@ioc.fiocruz.br (F.d.S.); 2 Laboratório de Flavivirus, Instituto Oswaldo Cruz, Fundação Oswaldo Cruz/FIOCRUZ, Rio de Janeiro 21040-900, Brazil; ritanog72@gmail.com; 3 Laboratório de Anatomia Patológica, Instituto Nacional de Infectologia, Fundação Oswaldo Cruz/FIOCRUZ, Rio de Janeiro 21040-900, Brazil; janice.coelho@ini.fiocruz.br (J.C.); francisco.rodrigues@ini.fiocruz.br (F.R.); 4 Laboratório Interdisciplinar de Pesquisas Médicas, Instituto Oswaldo Cruz, Fundação Oswaldo Cruz/FIOCRUZ, Rio de Janeiro 21040-900, Brazil; natgsalomao@gmail.com (N.S.); carollina.ceia@gmail.com (C.J.); 5 Laboratório de Ultraestrutura e Biologia Tecidual, Universidade do Estado do Rio de Janeiro/UERJ, Rio de Janeiro 20551-030, Brazil; jjcarv@gmail.com (J.d.C.); kissilarabelo91@gmail.com (K.R.); 6 Anatomia Patológica, Universidade Federal do Estado do Rio de Janeiro, Rio de Janeiro 20270-901, Brazil; rodrigopboliveira@gmail.com (R.B.-d.-O.); basiliopatologia@br.inter.net (C.B.-d.-O.)

**Keywords:** dengue 4, pregnancy, fetal death, cytokines, inflammatory mediators

## Abstract

Dengue virus (DENV) is an emerging virus involved in outbreaks in Brazil. The association between the virus and vertical transmission, with disorders in the placenta, has raised a worldwide concern. On the 29th gestational week, a pregnant woman presented severe complications due to a DENV infection leading to maternal and fetus death. Postmortem analysis of fetal organs demonstrated the presence of DENV using reverse transcriptase polymerase chain reaction (RT-PCR) in the fetal brain and DENV non-structural protein 3 (NS3) staining in placenta and several peripheral fetal tissues, such as the brain, liver, lungs, and spleen. Histological analysis of the placenta and fetal organs revealed different types of tissue abnormalities, which included inflammation, hemorrhage, edema, and necrosis in placenta and tissue disorganization in the fetus, such as spongiform parenchyma, microglial inflammation, steatosis, hyalinose arteriolar, inflammatory cells in the alveolar septa, and disorganization of the lymphoid follicle. Increased cellularity (macrophage, Hofbauer cells and TCD8+ lymphocytes) and up-regulation of inflammatory mediators such as IFN-γ, TNF-α, RANTES/CCL5, MCP1/CCL2, and VEGF/R2 were detected in the liver, lung, spleen, brain, and placenta, supporting placental and fetus peripheral tissues inflammation. Maternal infection leading to the production of those vascular mediators may alter the vascular permeability, facilitating the virus entry and tissue and barrier dysfunction.

## 1. Introduction

Dengue is a mosquito-borne viral disease endemic in many tropical and subtropical regions worldwide [1]. Approximately half of world’s population is at risk of infection by one of the four Dengue virus (DENV) serotypes [2]. In Brazil, dengue emerged as a public health problem after DENV-1 was introduced in 1986 [3]. After 32 years, four DENV serotypes are currently co-circulating [4].

Despite its first detection in Roraima 1981, which is north of Brazil [5], it was only in 2010 that DENV-4 was reintroduced [6] and spread to other states in the country, including Rio de Janeiro (RJ) in the Southeast region [7]. Due to the populations’ susceptibility to this newly introduced serotype, explosive epidemics in the country were a real threat and the impact of the DENV-4 emergence in an endemic region where the other three serotypes were circulating was unknown [8]. DENV-4 spread countrywide and caused explosive epidemics in the following years. In 2013, Brazil experienced an intense epidemic, with the co-circulation of the four serotypes and a total of 1,452,489 reported cases. RJ reported a total of 213,058 dengue cases, about 15% of the whole country [9]. In fact, DENV-4 was responsible for the highest number of cases in RJ and the metropolitan region was responsible for most cases in 2013.

The disease has a broad clinical spectrum, from asymptomatic and oligosymptomatic forms to severe conditions [10]. Currently, the World Health Organization (WHO) 2009 guidelines classify the illness as dengue without warning signs, dengue with warning signs, and severe dengue [11]. Severe dengue can be characterized by severe bleeding, severe organ involvement, and severe plasma leakage; most deaths are associated to this condition. In the absence of supportive care, severe dengue fatality can occur in approximately 4% of cases [12]. DENV-4 is known as a mild serotype and has been associated to severe and fatal cases in Brazil [8]. Histopathological analysis in dengue fatal cases has demonstrated alterations and/or inflammatory reactions in the liver, spleen, kidney, lung, heart, and central nervous system [13,14,15,16,17,18,19,20,21].

Pregnant women and neonates are considered to be at increased risk of a more severe disease [22,23]. Although there is no consensus regarding the effects of the disease on this vulnerable group, some studies indicate that vertical transmission may occur and cause serious consequences, such as preterm delivery and fetal death [24,25,26,27,28]. There is some evidence that the risk of severe dengue and hospitalization is higher among pregnant women compared to non-pregnant women [29] and that the maternal natural immunosuppression during pregnancy may favor the occurrence of a more severe infection [30]. Immunohistochemical analysis revealed a systemic involvement of infection with mononuclear cells targeted to all of the tissues analyzed. Assessment of local cytokine response showed increased levels of IFN-γ- and TNF-α-expressing cells in all tissues that evidenced a consistent pro-inflammatory induction and inflammatory mediators.

Maternal–fetal interface studies are still in need, as several agents, including arboviruses, can be transmitted from mother to her offspring, leading to a wide spectrum of outcomes [31]. Despite the efforts and studies worldwide [23,27,28,29,30,32,33,34,35,36,37,38,39,40,41], the burden of dengue during pregnancy on the mother/child pair is not fully understood, but shall be undoubtedly be taken seriously, especially where multiple arboviruses co-circulate, such as in Brazil [27]. Here, we sought to investigate multiple organs of a fetus from an abortion occurred in a DENV-4 infected pregnant woman during the outbreak in RJ in 2013.

## 2. Materials and Methods

### 2.1. Case

A 29-week-old pregnant woman, 36 years old, resident of Itaboraí, metropolitan region of RJ, started a febrile illness with vomiting, arthralgia, headache, and epigastria on 03/16/2013. She was admitted to a hospital in RJ on 03/17/2013, presenting a leukocyte count of 13,000/mm³, hematocrit of 33.7%, platelet count of 276,000/mm³, and a positive result for a DENV non-structural protein 1 (NS1) antigen. She was dismissed and requested to return within 48 h for a new evaluation. On 03/22/13, the patient returned to the health unit with pain in the lower limbs, vaginal bleeding, a leukocyte count of 14,000/mm³, a hematocrit of 34.3%, and a platelet count of 112,000 mm³. She was hospitalized in the intensive care unit (ICU). The following day, she had intense bleeding, blood pressure of 13 × 10, vomiting with blood, a leukocyte count of 445,000/mm³, a hematocrit of 26.5%, and a platelet count of 56,000/mm³. The ultrasound revealed a stillborn; vaginal delivery was performed. The fetus was detached from the placenta and an autopsy was performed. Fragments of liver, spleen, brain, lung, and placenta were collected and sent to the Flavivirus Laboratory, FIOCRUZ on 03/27/2013 for case investigation. After vaginal delivery, she remained in the ICU, but died 11 days later, on 04/02/13. No autopsy was performed at that time. The case was classified as Dengue with Complications (DCC), according the criteria established by the Brazilian Ministry of Health in 2000. DCC was established to define severe dengue cases that did not meet the 1997 WHO criteria for dengue hemorrhagic fever (DHF) and dengue shock syndrome (DSS).

### 2.2. Ethical Considerations

The samples used in this study were received as convenience samples at the Flavivirus Laboratory, Oswaldo Cruz Institute, FIOCRUZ, Regional Reference Center for Dengue, Yellow Fever, Chikungunya, and Zika for the Brazilian Ministry of Health. This study was approved by the Research Ethics Committee (CEP 274/05 and CAAE: 57221416.0.1001.5248) of the Oswaldo Cruz Foundation, Ministry of Health, Brazil.

### 2.3. Molecular Diagnosis, Histopathological Analysis, and Immunohistochemistry

Tissues samples from necropsy were paraffin-embedded, fixed in 10% formalin, cut (4 µm), deparaffinized in xylene and rehydrated with alcohol, as described elsewhere [18]. For the paraffin-embedded viral ribonucleic acid (RNA) extraction, three 5-μm slices of each fragment were used and submitted separately to the PureLink ™ FFPE RNA Isolation Kit (Invitrogen, Carlsbad, CA, USA). The reverse transcriptase polymerase chain reaction (RT-PCR) for DENV identification and serotyping was performed as described by Lanciotti et al. [42]. Tissues sections were stained with hematoxylin and eosin for histological examination and visualized using light microscopy (Olympus, Tokyo, Japan); digital images were obtained using Image Pro Plus software version 4.5 (Media Cybernetics, Rockville, MD, USA). For immunohistochemical studies, antigen retrieval was performed by heating the tissue in the presence of EnVision Flex target retrieval solution high pH (Dako, Palo Alto, CA, USA) or citrate buffer. Tissues were blocked for endogenous peroxidase with 3% hydrogen peroxidase in methanol and rinsed in Tris-HCl (pH 7.4). To reduce non-specific binding, sections were incubated for 30 min at room temperature. Samples were then incubated overnight at 4 °C with anti-DENV NS3 recombinant antibody [18], rabbit anti-human CD4 monoclonal antibody clone SP35 (Spring Bioscience, Pleasanton, CA, USA), mouse anti-human CD8 Clone C8/144B (Dako, Palo Alto, CA, USA), macrophage antibody CD68 clone EBM11 (Dako, Palo Alto, CA, USA), anti-MCP 1 monoclonal antibody (Novus Biologicals, Centennial, CO, USA), anti-TNF alpha antibody, Clone ab6671 (Abcam, MA, USA), goat IFN-γ (D-17) polyclonal antibody (Santa Cruz Biotechnology, Dallas, TX, USA), anti-RANTES antibody, and Clone ab189841 (Abcam, Cambridge, MA, USA); anti-VEGF/R2 (Spring Bioscience, Pleasanton, CA, USA). The next day, the sections were incubated with REVEAL COMPLEMENT secondary antibody (Spring Bioscience, Pleasanton, CA, USA) for 10 min and a REVEAL-HRP secondary antibody conjugate (Spring Bioscience, Pleasanton, CA, USA) for 15 min at room temperature. The reaction was revealed with diaminobenzidine (Spring Bioscience, Pleasanton, CA, USA), as chromogen and sections were counterstained on Harris hematoxylin (Dako, Palo Alto, CA, USA).

## 3. Results

**Figure 1:**
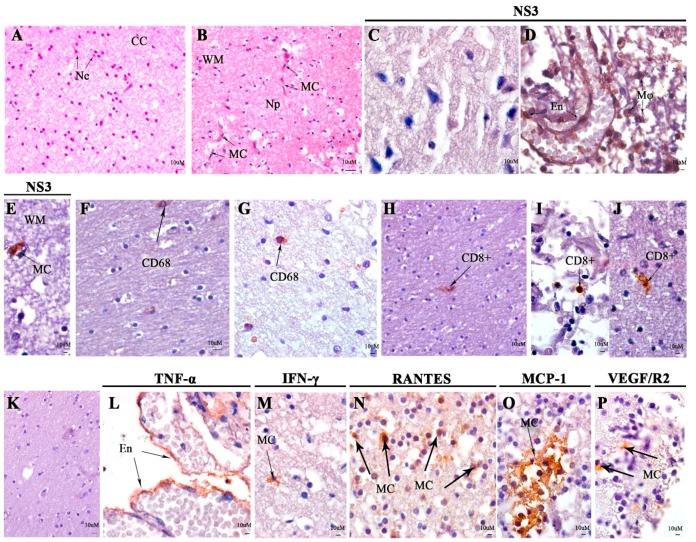
Histopathological and immunohistochemistry analysis of the fetal brain. (**A**) Brain of a non-DENV case presenting normal aspect: cerebral cortex (CC) and neurons (Ne). (**B**) White matter (WM) region of the stillborn brain presenting neuropile (Np) and microglial cells (MC). (**C**) DENV NS3 staining by immunohistochemistry in negative control. (**D**) DENV NS3 protein present in circulating macrophages (Mø) and endothelial cells (En) in blood vessels in the meningeal region and (**E**) microglial cells (MC) in WM. CD68 detection in negative control (**F**) and DENV case (**G**). CD8^+^ T cell detection in vessels in negative control (**H**), meningel region (**I**) and parenchyma (**J**) both in DENV cases. (**K**) Representative negative control non-DENV of cytokine and inflammatory mediators. (**L**) Endothelial cells (En) TNF-α expressing in DENV-case. IFN-γ (**M**), RANTES (**N**), MCP-1 (**O**), and VEGF/R2 (**P**) expressing in microglial cells (MC) from DENV-case parenchyma.

Fragments of the liver, lung, spleen, brain, and placenta were available and after viral RNA extraction, the RT-PCR detected DENV-4 infection only in the fetal brain. In the brain, an increase in microglial cells and glial cells was observed in the white matter region (Figure 1B,C). As expected, the brain tissue of a non-infected fetus (negative control) showed a pyramidal neuron layer and white matter with regular structures (Figure 1A). Meningeal thickening presented mononuclear inflammatory infiltrate and DENV NS3 protein was detected in endothelium, macrophages, and microglial cells in the white matter (Figure 1D,E). The inflammatory infiltrates were more diffuse in the white matter with the predominance of microglial cells positive for CD68^+^ (Figure 1G), while the control only showed CD68 staining in monocytes in the cerebral capillary (Figure 1H). CD8+ T cells were observed in the meningeal, pia mater, and cerebral parenchyma (Figure 1I,J). Microglial cells expressing RANTES, IFN-γ, MCP-1, and VEGF/R2 were identified in the spongiform parenchyma and TNF-α was detected in endothelial cells in the meninger’s vessels (Figure 1L–P).

**Figure 2:**
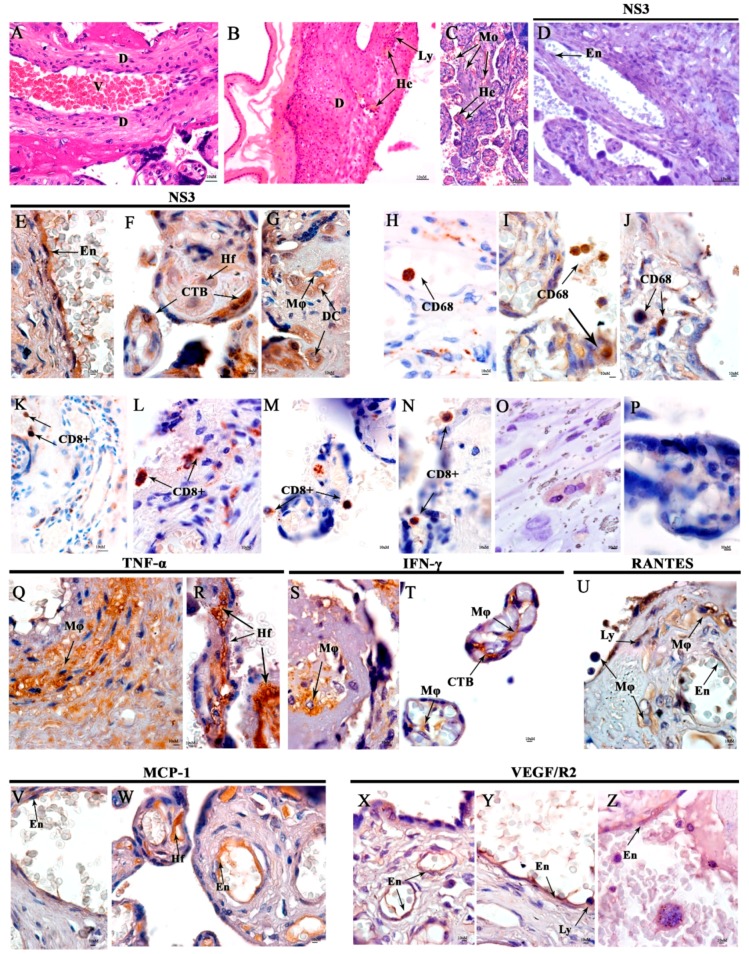
Histopathological and immunohistochemistry analysis of the placenta. (**A**) Non-DENV patient stained with HE and presenting normal maternal decidua (**D**) and blood vessels (V). (**B**) Maternal decidua (D) from the DENV case presenting focal hemorrhage and lymphocytic infiltrates. (**C**) Chorionic villus with mononuclear infiltrate (Mo) and haemorrhage (He) and also intervillus space in the DENV case. (**D**) Placenta control with no DENV NS3 protein detection. (**E**) DENV NS3 protein detection in endothelial cells (En) from the maternal vessel, (**F**) in the cytotrophoblasts (CTB) and Hofbauer cells (Hf), (**G**) in decidual cells (DC), and macrophages (Mø) in the infected placenta. (**H**) CD68 detection in negative control and in the DENV case (**I**,**J**). (**K**) CD8^+^ T cells detection in negative control and in the DENV case (**L**–**N**). (**O**,**P**) Representative negative control of cytokine and inflammatory mediators from a non-DENV case (**Q**,**R**) Expressing TNF-α in macrophages (Mø) and Hofbauer cells (Hf) in the DENV-case. (**S**) Expressing IFN-y in macrophages (Mø) in maternal region, (**T**) in Macrophages (Mø) and cytotrophoblasts (CTB) in chorionic villi. (**U**) Expressing RANTES in Macrophages (Mø), endothelial cells (En), and lymphocytes (Ly) in the maternal region. (**V**) Endothelial cells (En) expressing MCP-1 in the maternal region and, (**W**) endothelial cells (En), and Hofbauer cells (Hf), both in chorionic villi. (**X**–**Z**) Macrophages (Mø), endothelial cells (En) and lymphocytes (Ly) expressing VEGF/R2.

In the placenta, vacuolization around the maternal decidual cells associated to vascular areas containing mononuclear infiltrate and vascular congestion was observed. Hemorrhage and mononuclear infiltrate were observed in the chorionic villi (Figure 2B,C). In the maternal region, DENV NS3 protein was detected in the endothelial cells from the vessels, in the cytotrophoblasts cells, Hofbauer cells in chorionic villi, macrophages, and decidual cells (Figure 2E,G). Moreover, circulating macrophages and monocytes expressing CD68+ cells in the intervillous space and Hofbauer cells were detected (Figure 2I,J). In addition, in the maternal region, activated CD8+ T cells were detected in the chorionic villus (Figure 2L,O). Expression of TNF-α, IFN-γ, RANTES, and MCP-1 were observed in macrophages, Hofbauer cells, cytotrophoblasts, and endothelial cells. Furthermore, VEGF/R2 was detected in endothelium and lymphocytes (Figure 2Q,U). Controls exhibited normal chorionic villi syncytiotrophoblasts, cytotrophoblasts, and endothelial cells (Figure 2A).

**Figure 3:**
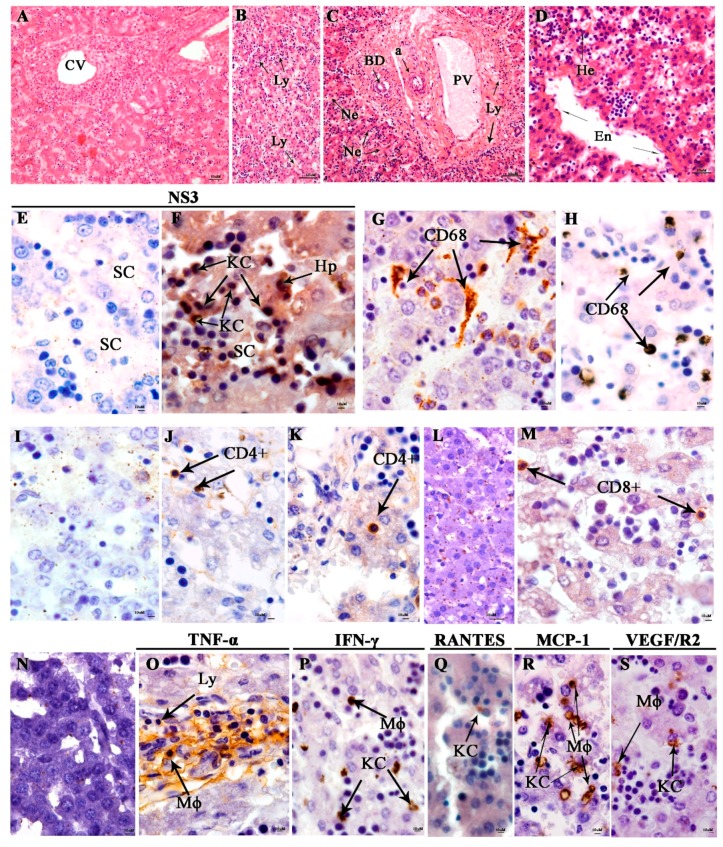
Histopathological and immunohistochemistry analysis of the liver. (**A**) Liver of a non-dengue case stained with HE, presenting a normal central vein (CV) aspect. Stillborn liver with lymphocytes infiltrate (Ly) in the lobular center (**B**), (**C**) area of necrotic hepatocytes (Ne), with normal bile duct (BD) and lymphocytes infiltrate (Ly) in the peri-portal vein (PV), (**D**) thickening of the endothelium (En) in the lobular center and presence of hemorrhage (He). (**E**) Sinusoids capillaries of the negative control without DENV NS3 staining. (**F**) DENV NS3 protein in Kupffer cells (HPC) and hepatocytes (Hp) near the sinusoids capillaries. CD68 cells staining in the control (**G**) and in the dengue case (**H**). No evidence of CD4^+^ (**I**) and CD8^+^ T cells (**L**) in the negative control. CD4^+^ (**J**,**K**) and CD8^+^ (**M**) T cells detection in the dengue case. (**N**) Representative negative control of cytokine and inflammatory mediators from a non-DENV case. (**O**) TNF-α expression in macrophages (Mø) and lymphocytes (Ly). (**P**,**R**,**S**) IFN-γ, MCP-1, and VEGF/R2 in macrophages (Mø) and Kupffer cells (HPC). (**Q**) RANTES expression in Kupffer cells (HPC).

The liver’s negative control showed normal parenchyma and regular central vein and portal space (Figure 3A). The stillborn liver showed a diffuse area of necrotic hepatocytes with mononuclear infiltrate, microesteatosis, macrosteatosis, hyperplasia of Kupffer cells, polyploidy, discrete area of lymphocyte infiltrate in the sinusoidal capillary, thickening of the endothelium in the central vein, and presence of edema (Figure 3B,D). DENV NS3 protein was detected in the Kupffer cells and hepatocytes (Figure 3F) and CD68+ was expressed the hyperplasic Kupffer cells and circulating macrophages (Figure 3G,H). CD8^+^ and CD4^+^ T cells were identified inside the hepatocyte’s cytoplasm (Figure 3J,K). Kupffer cells and endothelial cells were expressing TNF-α in macrophages, lymphocytes (Figure 3O), and Kupffer cells expressing IFN-γ and RANTES (Figure 3P,Q). Circulating Kupffer cells and macrophages expressed MCP-1 and VEGF/R2 in the sinusoidal capillaries (Figure 3R,S). The control liver tissue showed low density of positive cells (Figure 3N).

**Figure 4:**
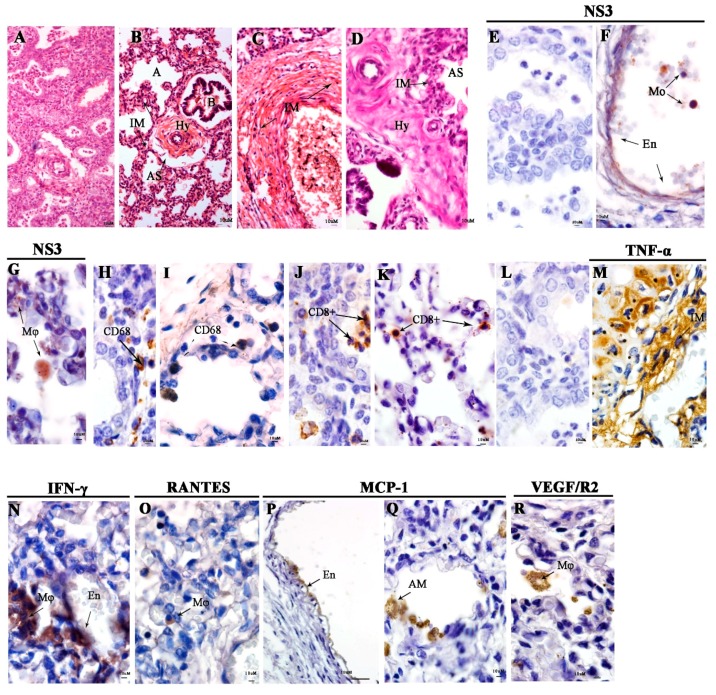
Histopathological and immunohistochemistry analysis of the lung. (**A**) Lung of a non-dengue case. In the stillborn lung, we observed the (**B**) presence of mononuclear infiltrates (IM) around the bronchus and hyalinosis (Hy). (**C**) Mononuclear infiltrates (IM) in the muscular tunica and (**D**) focal area of alveolar hyalinosis (Hy) with mononuclear infiltrate (IM). (**E**) Negative control without DENV NS3 staining. (**F**) Detection of DENV NS3 in monocytes (Mo) and endothelial cells (En) in vessels and (**G**) macrophage (Mø), in the alveolar space. (**H**,**I**) Presence of CD68 and (**J**,**K**) CD8^+^ T cells in the alveolar space. (**L**) Representative negative control of cytokine and inflammatory mediators from a non-DENV case. (**M**) TNF-α in mononuclear infiltrate (IM) (**N**) IFN-γ in macrophages and endothelial cells (En), (**O**) RANTES in macrophages (Mø), (**P**,**Q**) MCP-1 in endothelial cells (En), alveolar macrophages (AM), and (**R**) VEGF/R2 in macrophages (Mø).

In the lung, we observed an increase of mononuclear infiltrate around the bronchus, hyalinosis, and mononuclear infiltrate in the muscular tunic, in the pulmonary artery layers, and focal area of alveolar thickening (Ht) (Figure 4B,D). DENV NS3 protein was detected in the endothelial cells, monocytes, and macrophages in the alveolar septum (Figure 4F,G). Cells expressing CD68^+^ and CD8^+^ in the alveolar septum were observed (Figure 4I,K). TNF-α, IFN-γ, RANTES, MCP-1, VEGF/R2, and MCP-1 were detected in macrophages and endothelial cells in the pulmonary vein (Figure 4M,R). The control lung tissue showed a low density of positive cells (Figure 4L).

**Figure 5:**
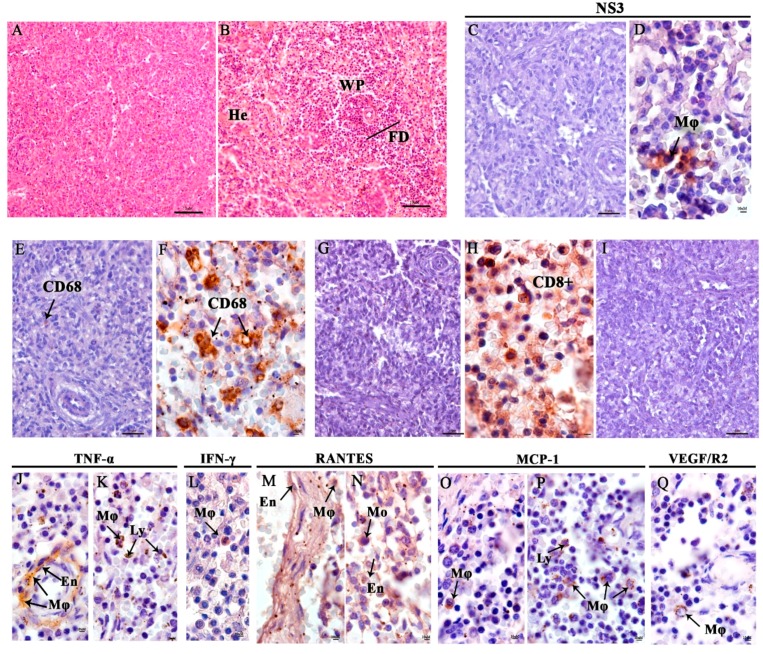
Histopathological and immunohistochemistry analysis of the spleen. (**A**) Spleen of a non-dengue case. (**B**) In the stillborn spleen, hemorrhage (He) in the red and white pulps, and follicle disorganization (FD) in the white pulp, were observed. (**C**) Negative control without NS3 staining. (**D**) Detection of DENV NS3 protein in macrophage (Mø). (**E**,**F**) Presence of CD68. Negative control without TCD8^+^ cells (**G**). (**H**) Presence of T CD8^+^ cells in red pulp. (**I**) Representative negative control of cytokine and inflammatory mediators from a non-DENV case. (**J**,**K**) TNF-α in macrophages (Mø), endothelial cells (En), and lymphocytes (Ly), (**L**) IFN-y in macrophages, (**M**,**N**) RANTES in macrophages (Mø), endothelial cells (En) and monocytes (Mo), (**O**,**P**) MCP-1 in macrophages (Mø), lymphocytes (Ly), and (**Q**) VEGF/R2 only in macrophages (Mø).

In the spleen, vascular congestion and follicle disorganization in the white pulp were observed (Figure 5B). DENV NS3 was detected in splenic macrophage in the follicle in germinative center of the white pulp (Figure 5D,E). CD68^+^ expression was observed in activated macrophage cells that presented degenerated cytoplasm (Figure 5F) and CD8^+^ T cells in the red pulp area (Figure 5H). TNF-α and IFN-γ were expressed in macrophages in germinal center areas in the white pulp, but around the central arteriole and splenic macrophage in the red pulp (Figure 5J,L). RANTES was also expressed in endothelial cells and macrophages with expansive cytoplasm (Figure 5M,N) and vascular mediators MCP-1 and VEGF/R2 were present in the white pulp (Figure 5O,Q). The control spleen tissue showed low density of positive cells (Figure 5I).

## 4. Discussion

The risk of dengue infection in pregnant women is still inconclusive and controversial [43]. However, some authors consider pregnant women more likely to progress to more severe forms of the disease [23,27,29,30,36,41]. A recent study reported that DENV vertical transmission rates might vary between 18.5% and 22.7%. Moreover, mother-to-child DENV transmission occurs both at the beginning and at the end of the pregnancy, being more frequent when maternal dengue occurs late during gestation, near delivery [24]. Evidences suggest that symptomatic dengue fever during pregnancy may be associated with adverse fetal outcomes, such as miscarriage, stillbirth, prematurity, and low birth weight [23,40,44,45,46]. Here, alterations such as inflammation, hemorrhage, edema, and necrosis were found in the placenta, brain, spleen, liver, and fetal lung. In addition, the presence of DENV-specific NS3 protein indicates viral replication in these tissues. Similar changes caused by DENV were reported in adult fatal cases [15,18,47].

Nunes et al. [48] analyzed the placenta from a DENV-2 case and reported areas of chorioangiosis and placental villous, as well as hypotrophy and infiltration of macrophages in the adventitia tunica of the artery in the umbilical cord. Similarly, Ribeiro et al. [49] analyzed placentas during dengue epidemics in Rio de Janeiro and identified signs of hypoxia, choriodeciduitis, deciduitis, and intervilositis, corroborating the observations here. However, Rabelo et al. [50] also reported the presence of large diffuse areas of fibrinoid necrosis in the maternal decidua from a zika vertical transmission, with evidenced diffuse edema, fibrosis, vascular endothelial thickening, degeneration, vascular congestion, and focal areas of mononuclear cells or perivascular inflammatory infiltrates.

The viral transmission to the fetal-placental tissues can occur through the maternal vascular endothelium to the endovascular extra viral trophoblasts; by infected maternal blood macrophages, which transmit the infection to placental trophoblasts, and by paracellular routes from maternal blood to the fetal capillaries [51,52]. Moreover, it has been reported previously that the potential mechanisms by which a maternal infection may result in fetal death outcome includes direct fetal infection and organ damage, placental infection resulting in decreased transmission of nutrients, and oxygen and maternal illness with increased production of cytokines and chemokines [53].

The role of cytokines in the pathogenesis of dengue severity has been demonstrated as a consensus that inflammatory response associated with deregulated cytokine production is critical for development of severe cases, besides the virus-mediated pathogenesis. The significant increase in several soluble inflammatory mediators, “cytokine storm”, is a well-known event and is present in the most severe forms of the disease [54]. Furthermore, activated CD4^+^ and CD8^+^ T cells generated in response to DENV infection, may produce cytokines and inflammatory mediators, lyse infected target cells for viral control, and tissue injury [55,56,57].

TNF-α is a pleiotropic cytokine that regulates many physiological and pathological functions such as cell survival, apoptosis, migration, and inflammation [58]. In fact, TNFα is associated with dengue severity in patients [59,60] inducing vascular permeability, as well as metalloproteinases [61] that would act on endothelial cells. Importantly, pregnancy is associated with a Th2 response with a decrease of Th1 induction and, the balance of pro and anti-inflammatory cytokines is critical for implantation, placental development, and pregnancy outcome [62]. However, a cytokine imbalance could be responsible for alterations in the placental environment and be involved in unexplained recurrent miscarriages. Therefore, TNF-α plays an essential role during pregnancy, especially in the trophoblast turnover and renewal. At the same time, TNF might be detrimental to pregnancy, causing complications such as miscarriage and preeclampsia [63]. RANTES is important in the recruitment of leukocytes to inflamed sites [64] and previous studies found low circulating levels of RANTES in the blood of dengue patients, while high expression was found in the hepatic tissue of fatal cases [65,66]. In severe dengue, the increased expression of RANTES, MCP-1, and VEGF/R2, associated with increased permeability of endothelial cells may be indicative of a dysfunction of the blood-brain and placental barriers, increasing viral dissemination and inflammation [67,68,69,70,71].

## 5. Conclusions

The production of vascular mediators during the maternal dengue infection may alter the vascular permeability, facilitating virus entry, and barrier dysfunction, mainly inflaming the placenta and fetal brain. The infection exacerbation occurs, concomitantly, with the viral spread to the peripheral regions, causing infection in DENV target organs such as the liver, lung, and spleen, which play a role on the uptake circulation of infected cells, leading to a widespread infection in the fetus. Despite the reports suggesting that stillbirth might result from direct viral transmission to the fetus, the role of the maternal infection in this outcome remains to be fully elucidated [45]. This study has some limitations and those include the quality of the record resulting in the lack of some clinical information and the fact that, after the mother’s death, no autopsy was performed, therefore, no additional tissues were available, besides the placenta.
